# (*E*)-2-[(2-Amino-4,5-dibromo­phen­yl)imino­meth­yl]-6-methoxy­phenol

**DOI:** 10.1107/S1600536808044164

**Published:** 2009-01-08

**Authors:** Zhan-Xian Li, Hui Yang, Ming Yu, Qiu-Zhi Shi, Ming-Ming Yu

**Affiliations:** a Department of Chemistry, Zhengzhou University, Zhengzhou 450001, People’s Republic of China

## Abstract

The title compound, C_14_H_12_Br_2_N_2_O_2_, was prepared from the condensation of 4,5-dibromo-1,2-phenyl­enediamine and 2-hydr­oxy-3-methoxy­benzaldehyde in methanol. The N=C double bond shows a *trans* conformation and the dihedral angle between the aromatic ring planes is 5.9 (4)°. In the crystal structure, there are intra­molecular O—H⋯N and N—H⋯N and inter­molecular N—H⋯O hydrogen bonds, the latter resulting in inversion dimers.

## Related literature

For related literature on the design of ligands for polynuclear coordination complexes with novel magnetic properties, see: Fernández *et al.* (2001 ▶); Pardo *et al.* (2003 ▶); Yu *et al.* (2007 ▶). For the synthesis and structure of a related compound, see: Xia *et al.* (2007 ▶). 
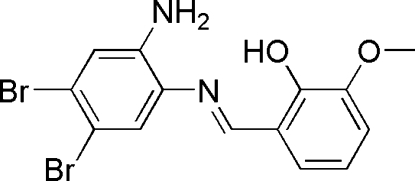


## Experimental

### 

#### Crystal data

C_14_H_12_Br_2_N_2_O_2_
*M* *_r_* = 400.08
Triclinic, 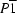

*a* = 6.9500 (4) Å
*b* = 7.4383 (4) Å
*c* = 14.7877 (10) Å
α = 100.351 (5)°
β = 97.218 (5)°
γ = 109.967 (3)°
*V* = 692.21 (8) Å^3^
*Z* = 2
Mo *K*α radiation
μ = 5.86 mm^−1^
*T* = 292 (3) K
0.50 × 0.40 × 0.22 mm

#### Data collection

Bruker SMART 1K CCD area-detector diffractometer
Absorption correction: multi-scan (*SADABS*; Bruker, 2000 ▶) *T*_min_ = 0.072, *T*_max_ = 0.275
6557 measured reflections
3154 independent reflections
2695 reflections with *I* > 2σ(*I*)
*R*_int_ = 0.018

#### Refinement

*R*[*F* ^2^ > 2σ(*F* ^2^)] = 0.025
*wR*(*F* ^2^) = 0.071
*S* = 1.02
3154 reflections
190 parameters
H atoms treated by a mixture of independent and constrained refinement
Δρ_max_ = 0.60 e Å^−3^
Δρ_min_ = −0.46 e Å^−3^

### 

Data collection: *SMART* (Bruker, 2000 ▶); cell refinement: *SAINT* (Bruker, 2000 ▶); data reduction: *SAINT*; program(s) used to solve structure: *SHELXTL* (Sheldrick, 2008 ▶); program(s) used to refine structure: *SHELXTL*; molecular graphics: *SHELXTL*; software used to prepare material for publication: *SHELXTL*.

## Supplementary Material

Crystal structure: contains datablocks I, global. DOI: 10.1107/S1600536808044164/hg2454sup1.cif

Structure factors: contains datablocks I. DOI: 10.1107/S1600536808044164/hg2454Isup2.hkl

Additional supplementary materials:  crystallographic information; 3D view; checkCIF report

## Figures and Tables

**Table 1:** Hydrogen-bond geometry (Å, °)

*D*—H⋯*A*	*D*—H	H⋯*A*	*D*⋯*A*	*D*—H⋯*A*
O2—H2⋯N1	0.82	1.88	2.608 (2)	147
N2—H2*A*⋯N1	0.84 (3)	2.39 (3)	2.756 (3)	107 (2)
N2—H2*B*⋯O1^i^	0.81 (3)	2.30 (3)	3.114 (3)	174.19

Symmetry code: (i) 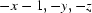.

**Fig. 1.**
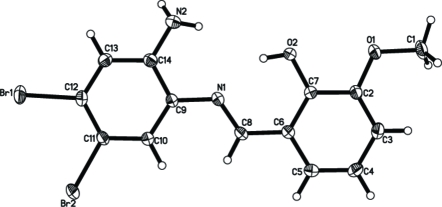
A view of complex (I), showing 30% probability displacement ellipsoids and the atom-numbering scheme.

**Fig. 2.**
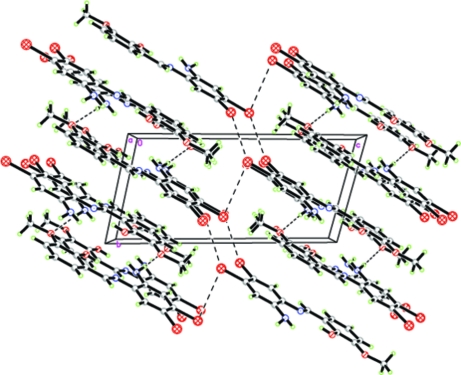
The crystal packing of title compound, viewed along the a axis. Hydrogen bonds are shown as dashed lines.

## supplementary crystallographic information

### Comment

The design and synthesis of new ligands with the potential for forming polynuclear coordination complexes with novel magnetic properties is of current research interest (Pardo, *et al.*, 2003; Yu, *et al.*, 2007; Fernández,*et al.*, 2001) and we report here the synthesis and crystal structure of the title complex (I).

The molecular structure of title compound is showing in Fig. 1. In the structure, there are intramolecular O—H···N, N—H···N and intermolecular N—H···O hydrogen bonds in the crystal lattice.

### Experimental

The title compound was synthesized according to modified reported methods (Xia, *et al.*, 2007). 1 mmol (265.9 mg) 4,5-dibromo-1,2-phenylenediamine and 1.1 mmol (167.4 mg) 2-hydroxy-3-methoxybenzaldehyde were dissolved in 30 ml solution of methanol, then refluxed for 2 h. The solution was cooled and filtered. Crystals suitable for X-ray diffraction analysis were obtained by slow evaporation at room temperature for 10 days.

### Refinement

All H atoms were placed in geometrically calculated positions with C—H = 0.96 Å for methyl H atoms, C—H = 0.93 Å for aromatic H atoms and 0.82 Å for N—H. H atoms and were refined isotropic with *U*_iso_(H) = 1.2*U*_eq_(C) of parent atom using a riding model. The H atom of the hydroxy group was located from difference maps and refined with a distance restraint O—H = 0.82 (1) Å. *U*_iso_(H) = 1.2*U*_eq_(O).

### Crystal data

**Table:**

C_14_H_12_Br_2_N_2_O_2_	*Z* = 2
*M**_r_* = 400.08	*F*(000) = 392
Triclinic, *P*1	*D*_x_ = 1.919 Mg m^−^^3^
Hall symbol: -P 1	Mo *K*α radiation, λ = 0.71073 Å
*a* = 6.9500 (4) Å	Cell parameters from 3154 reflections
*b* = 7.4383 (4) Å	θ = 1.4–27.6°
*c* = 14.7877 (10) Å	µ = 5.86 mm^−^^1^
α = 100.351 (5)°	*T* = 292 K
β = 97.218 (5)°	Block, colourless
γ = 109.967 (3)°	0.50 × 0.40 × 0.22 mm
*V* = 692.21 (8) Å^3^	

### Data collection

**Table:**

Bruker SMART 1K CCD area-detector diffractometer	3154 independent reflections
Radiation source: fine-focus sealed tube	2695 reflections with *I* > 2σ(*I*)
graphite	*R*_int_ = 0.018
φ and ω scans	θ_max_ = 27.6°, θ_min_ = 1.4°
Absorption correction: multi-scan (*SADABS*; Bruker, 2000)	*h* = −9→8
*T*_min_ = 0.072, *T*_max_ = 0.275	*k* = −9→9
6557 measured reflections	*l* = −12→19

### Refinement

**Table:**

Refinement on *F*^2^	Secondary atom site location: difference Fourier map
Least-squares matrix: full	Hydrogen site location: inferred from neighbouring sites
*R*[*F*^2^ > 2σ(*F*^2^)] = 0.025	H atoms treated by a mixture of independent and constrained refinement
*wR*(*F*^2^) = 0.071	*w* = 1/[σ^2^(*F*_o_^2^) + (0.0379*P*)^2^ + 0.3429*P*] where *P* = (*F*_o_^2^ + 2*F*_c_^2^)/3
*S* = 1.02	(Δ/σ)_max_ < 0.001
3154 reflections	Δρ_max_ = 0.60 e Å^−^^3^
190 parameters	Δρ_min_ = −0.46 e Å^−^^3^
0 restraints	Extinction correction: *SHELXTL* (Sheldrick, 2008), Fc^*^=kFc[1+0.001xFc^2^λ^3^/sin(2θ)]^-1/4^
Primary atom site location: structure-invariant direct methods	Extinction coefficient: 0.0093 (11)

### Special details

**Table:**

Geometry. All e.s.d.'s (except the e.s.d. in the dihedral angle between two l.s. planes) are estimated using the full covariance matrix. The cell e.s.d.'s are taken into account individually in the estimation of e.s.d.'s in distances, angles and torsion angles; correlations between e.s.d.'s in cell parameters are only used when they are defined by crystal symmetry. An approximate (isotropic) treatment of cell e.s.d.'s is used for estimating e.s.d.'s involving l.s. planes.
Refinement. Refinement of *F*^2^ against ALL reflections. The weighted *R*-factor *wR* and goodness of fit *S* are based on *F*^2^, conventional *R*-factors *R* are based on *F*, with *F* set to zero for negative *F*^2^. The threshold expression of *F*^2^ > σ(*F*^2^) is used only for calculating *R*-factors(gt) *etc*. and is not relevant to the choice of reflections for refinement. *R*-factors based on *F*^2^ are statistically about twice as large as those based on *F*, and *R*- factors based on ALL data will be even larger.

### Fractional atomic coordinates and isotropic or equivalent isotropic displacement parameters (Å^2^)

**Table:**

	*x*	*y*	*z*	*U*_iso_*/*U*_eq_	
Br1	0.04544 (4)	0.75243 (4)	0.476886 (17)	0.05025 (11)	
Br2	0.48830 (4)	0.77615 (4)	0.397735 (17)	0.04846 (10)	
O2	−0.2559 (2)	0.1156 (3)	−0.08390 (11)	0.0429 (4)	
H2	−0.2248	0.1805	−0.0295	0.064*	
O1	−0.3008 (3)	−0.0863 (3)	−0.25537 (11)	0.0458 (4)	
N1	−0.0125 (3)	0.3290 (3)	0.07626 (12)	0.0310 (4)	
C9	0.0126 (3)	0.4297 (3)	0.17005 (14)	0.0287 (4)	
C5	0.2944 (3)	0.2066 (4)	−0.09082 (17)	0.0378 (5)	
H5	0.4265	0.2712	−0.0525	0.045*	
N2	−0.3662 (3)	0.2994 (3)	0.14823 (17)	0.0429 (5)	
C2	−0.0994 (3)	0.0097 (3)	−0.20552 (14)	0.0318 (4)	
C8	0.1432 (3)	0.3179 (3)	0.04022 (15)	0.0334 (5)	
H8	0.2770	0.3794	0.0771	0.040*	
C14	−0.1716 (3)	0.4098 (3)	0.20455 (15)	0.0314 (4)	
C6	0.1178 (3)	0.2131 (3)	−0.05579 (14)	0.0298 (4)	
C10	0.2057 (3)	0.5418 (3)	0.22926 (15)	0.0316 (4)	
H10	0.3274	0.5541	0.2067	0.038*	
C7	−0.0805 (3)	0.1144 (3)	−0.11378 (14)	0.0298 (4)	
C13	−0.1544 (3)	0.5092 (3)	0.29647 (16)	0.0351 (5)	
H13	−0.2750	0.5003	0.3195	0.042*	
C3	0.0768 (4)	0.0078 (3)	−0.23831 (16)	0.0375 (5)	
H3	0.0639	−0.0598	−0.2995	0.045*	
C1	−0.3285 (4)	−0.1801 (4)	−0.35196 (16)	0.0471 (6)	
H1A	−0.4751	−0.2415	−0.3792	0.071*	
H1B	−0.2670	−0.2781	−0.3566	0.071*	
H1C	−0.2621	−0.0836	−0.3849	0.071*	
C11	0.2199 (3)	0.6352 (3)	0.32091 (15)	0.0320 (4)	
C12	0.0382 (4)	0.6204 (3)	0.35391 (15)	0.0329 (4)	
C4	0.2745 (4)	0.1064 (4)	−0.18074 (17)	0.0403 (5)	
H4	0.3927	0.1039	−0.2035	0.048*	
H2A	−0.360 (4)	0.212 (4)	0.105 (2)	0.043 (7)*	
H2B	−0.455 (5)	0.251 (4)	0.177 (2)	0.049 (8)*	

### Atomic displacement parameters (Å^2^)

**Table:**

	*U*^11^	*U*^22^	*U*^33^	*U*^12^	*U*^13^	*U*^23^
Br1	0.05665 (18)	0.04947 (16)	0.03545 (15)	0.01522 (13)	0.01513 (11)	−0.00727 (10)
Br2	0.03535 (14)	0.06002 (18)	0.03276 (14)	0.01257 (11)	−0.00551 (10)	−0.01218 (11)
O2	0.0239 (7)	0.0630 (11)	0.0291 (8)	0.0100 (7)	0.0034 (6)	−0.0066 (7)
O1	0.0331 (8)	0.0600 (11)	0.0258 (8)	0.0038 (8)	0.0011 (6)	−0.0050 (7)
N1	0.0293 (9)	0.0321 (9)	0.0252 (8)	0.0078 (7)	0.0017 (7)	0.0010 (7)
C9	0.0290 (10)	0.0287 (9)	0.0243 (9)	0.0093 (8)	0.0021 (8)	0.0015 (8)
C5	0.0263 (10)	0.0465 (12)	0.0367 (12)	0.0122 (10)	0.0043 (9)	0.0047 (10)
N2	0.0267 (10)	0.0477 (12)	0.0423 (12)	0.0055 (9)	0.0046 (9)	−0.0001 (10)
C2	0.0313 (10)	0.0340 (10)	0.0246 (10)	0.0078 (9)	0.0036 (8)	0.0040 (8)
C8	0.0266 (10)	0.0377 (11)	0.0286 (11)	0.0094 (9)	−0.0016 (8)	−0.0001 (8)
C14	0.0290 (10)	0.0294 (10)	0.0332 (11)	0.0093 (8)	0.0040 (8)	0.0059 (8)
C6	0.0273 (10)	0.0325 (10)	0.0270 (10)	0.0101 (8)	0.0031 (8)	0.0042 (8)
C10	0.0279 (10)	0.0339 (10)	0.0285 (10)	0.0103 (9)	0.0042 (8)	0.0003 (8)
C7	0.0264 (10)	0.0346 (10)	0.0263 (10)	0.0099 (8)	0.0052 (8)	0.0049 (8)
C13	0.0305 (11)	0.0338 (10)	0.0389 (12)	0.0106 (9)	0.0108 (9)	0.0039 (9)
C3	0.0444 (13)	0.0399 (12)	0.0285 (11)	0.0164 (10)	0.0110 (9)	0.0048 (9)
C1	0.0529 (15)	0.0477 (14)	0.0236 (11)	0.0061 (12)	−0.0004 (10)	−0.0025 (10)
C11	0.0305 (10)	0.0312 (10)	0.0275 (10)	0.0095 (8)	−0.0008 (8)	−0.0011 (8)
C12	0.0396 (11)	0.0299 (10)	0.0275 (10)	0.0137 (9)	0.0075 (8)	0.0010 (8)
C4	0.0329 (11)	0.0523 (14)	0.0405 (13)	0.0196 (11)	0.0141 (10)	0.0107 (10)

### Geometric parameters (Å, °)

**Table:**

Br1—C12	1.891 (2)	C2—C7	1.404 (3)
Br2—C11	1.890 (2)	C8—C6	1.449 (3)
O2—C7	1.350 (3)	C8—H8	0.9300
O2—H2	0.8200	C14—C13	1.395 (3)
O1—C2	1.372 (3)	C6—C7	1.400 (3)
O1—C1	1.430 (3)	C10—C11	1.382 (3)
N1—C8	1.285 (3)	C10—H10	0.9300
N1—C9	1.411 (3)	C13—C12	1.379 (3)
C9—C10	1.393 (3)	C13—H13	0.9300
C9—C14	1.408 (3)	C3—C4	1.394 (3)
C5—C4	1.368 (3)	C3—H3	0.9300
C5—C6	1.403 (3)	C1—H1A	0.9600
C5—H5	0.9300	C1—H1B	0.9600
N2—C14	1.382 (3)	C1—H1C	0.9600
N2—H2A	0.84 (3)	C11—C12	1.388 (3)
N2—H2B	0.81 (3)	C4—H4	0.9300
C2—C3	1.376 (3)		
			
C7—O2—H2	109.5	C9—C10—H10	119.3
C2—O1—C1	117.15 (19)	O2—C7—C6	121.88 (18)
C8—N1—C9	122.27 (18)	O2—C7—C2	118.60 (18)
C10—C9—C14	119.32 (18)	C6—C7—C2	119.52 (19)
C10—C9—N1	124.08 (19)	C12—C13—C14	121.3 (2)
C14—C9—N1	116.59 (18)	C12—C13—H13	119.4
C4—C5—C6	120.7 (2)	C14—C13—H13	119.4
C4—C5—H5	119.6	C2—C3—C4	120.7 (2)
C6—C5—H5	119.6	C2—C3—H3	119.7
C14—N2—H2A	111.2 (19)	C4—C3—H3	119.7
C14—N2—H2B	114 (2)	O1—C1—H1A	109.5
H2A—N2—H2B	111 (3)	O1—C1—H1B	109.5
O1—C2—C3	125.3 (2)	H1A—C1—H1B	109.5
O1—C2—C7	114.79 (19)	O1—C1—H1C	109.5
C3—C2—C7	119.9 (2)	H1A—C1—H1C	109.5
N1—C8—C6	122.43 (19)	H1B—C1—H1C	109.5
N1—C8—H8	118.8	C10—C11—C12	119.28 (19)
C6—C8—H8	118.8	C10—C11—Br2	118.48 (16)
N2—C14—C13	120.2 (2)	C12—C11—Br2	122.23 (16)
N2—C14—C9	121.2 (2)	C13—C12—C11	120.21 (19)
C13—C14—C9	118.57 (19)	C13—C12—Br1	118.15 (16)
C7—C6—C5	119.27 (19)	C11—C12—Br1	121.63 (16)
C7—C6—C8	121.15 (19)	C5—C4—C3	119.9 (2)
C5—C6—C8	119.57 (19)	C5—C4—H4	120.0
C11—C10—C9	121.3 (2)	C3—C4—H4	120.0
C11—C10—H10	119.3		

### Hydrogen-bond geometry (Å, °)

**Table:**

*D*—H···*A*	*D*—H	H···*A*	*D*···*A*	*D*—H···*A*
O2—H2···N1	0.82	1.88	2.608 (2)	147
N2—H2A···N1	0.84 (3)	2.39 (3)	2.756 (3)	107 (2)
N2—H2B···O1^i^	0.81 (3)	2.30 (3)	3.114 (3)	174.19

Symmetry codes: (i) −*x*−1, −*y*, −*z*.
